# Plasmonic and bi-piezoelectric enhanced photocatalysis using PVDF/ZnO/Au nanobrush

**DOI:** 10.1515/nanoph-2022-0194

**Published:** 2022-06-10

**Authors:** Xiaofei Zhao, Zhen Li, Jing Yu, Chonghui Li, Shicai Xu, Fengrui Li, Chentao Zhang, Baoyuan Man, Chao Zhang

**Affiliations:** School of Physics and Electronics, Shandong Normal University, Jinan 250014, China; College of Physics and Electronic Information, Dezhou University, Dezhou 253023, China; Department of Instrumental and Electrical Engineering, Xiamen University, Xiamen 361102, China

**Keywords:** bi-piezoelectric, nanobrush, photocatalysis, plasmonic, ZnO

## Abstract

The photocatalytic degradation, as an environmental-friendly technology, has great significance for cost-effective and efficient catalysis processes, wherein piezo-photocatalysis can significantly increase the catalytic degradation rate using both solar and mechanical energy. Here, a ternary heterostructure PVDF/ZnO/Au (PZA) nanobrush photocatalyst with high piezo-photocatalytic efficiency was presented via low-temperature hydrothermal and chemical reduction methods. Under both solar and mechanical energy, the current response and degradation rate of the as-synthesized PZA nanobrush all increase significantly compared with that under solar alone and under mechanical energy alone, and the excellent recyclability is investigated. It is found that the PZA nanobrush with ultrasonic-assisted piezo-photocatalysis completely degrade MO of 20 mg/L in 60 min, which exhibits greater enhancement of photocatalytic activity than with stirring-assisted piezo-photocatalysis due to higher power. The high piezo-photocatalytic activity of PZA nanobrush is attributed to the surface plasmon resonance (SPR) coupling of Au and built-in electric field originating from the ZnO and PVDF, which can increase the absorption of visible light, promote the charge transfer and separation of photogenerated electrons/holes. This work introduces the SPR and bipiezotronic effect to improve plasmonic photocatalysis with PZA heterostructures, which offers a new solution in green technologies to design high-performance catalysts for the environmental remediation.

## 1 Introduction

In recent years, the environmental pollution has received extensive attention worldwide owing to the rapid development of the industry and extensive use of chemical and biological reagent. The photocatalytic degradation is one of the most potential technology in solving the environmental problem especially wastewater treatment [1–3]. The solar, as clean and renewable energy, can degrade organic pollutants in aqueous solution into nontoxic molecules, having considerable potentials in environmental improvement [4, 5]. Nowadays, various semiconducting photocatalysts with high photocatalytic efficiency were widely investigated such as ZnO, TiO_2_, SnO, and CdS nanostructures [6–9]. Among them, ZnO nanorods (NRs), an n-type compound semiconductor, has been recognized as an efficient and ideal candidate in degrading organic pollutants due to the nontoxicity, cost-effective and large exciton binding energy (∼60 meV) [10–12]. However, its wide bandgap (∼3.2 eV) [13, 14] and high photocorrosion [15] result in low absorbance of visible light and poor photo-stability, which restrict its practical applications. In order to resolve the problem, tremendous efforts have been devoted to enhancing the photocatalytic activity of ZnO-based photocatalysts. For example, Dutta et al. successfully synthesized Mn-doped ZnO photocatalysts by replacing Zn^2+^ by Mn^2+^, showing more efficient photocatalytic effect than bare ZnO [16]. Xu et al. decorated the surface of ZnO with Ag nanoparticles (NPs) to improve photocatalytic efficiency deriving from the uniformly dispersed AgNPs and surface oxygen vacancies on ZnO NPs [17]. Liu et al. enhanced catalytic activity of ZnO NRs via harvesting light and mechanical energy simultaneously based on the cooperative effect of piezo-photocatalysis, which is much higher than that of the piezocatalysis only or photocatalysis only [18]. Whereas, the method by doping other element into ZnO may cause poor stability, especially for a long-time photocatalytic degradation. In contrast, coupling noble metals (such as Au, Ag, Pt) nanocrystals with ZnO can increase the absorption of visible light and improve the utilization of solar light owing to the effective surface plasmon resonance (SPR) effect. Another efficient approach is to enhance the built-in electric field to accelerate photoexcited carrier separation and migration utilizing piezoelectric properties of ZnO nanomaterials. The two promising strategies can greatly improve the photocatalytic efficiency and overcome the problem of poor stability.

On the other hand, many researches have devoted to enhance the piezoelectric effect of ZnO through integrating other piezoelectric materials. For instance, Lu et al. combined ZnO with Polyvinylidene fluoride (PVDF) film to cause bi-piezoelectric integration effect to enhance photocatalytic activity under low density flow water energy [19]. Kalarikkal et al. proposed BaTiO_3_/ZnO heterostructure to degrade methylene blue (MB) dye under UV irradiation, which exhibits improved photocatalytic activity [20]. However, these photocatalyst were based on single-layer planar structure with limited exposed surface. However, these projects were only based on piezoelectric field-assisted photocatalysis, which had limited utilization of solar light. Thus, it is highly expected to seek more effective photocatalyst to improve catalytic performances.

In this work, a new type of multilayer PVDF/ZnO/Au (PZA) nanobrush is developed via a simple chemical reduction/hydrothermal route to enhance sunlight photocatalytic degradation performances with the SPR effect of Au and bi-piezoelectric field of ZnO and PVDF. The PVDF was presented with electrostatic spinning to form nanofiber structure, which can enhance its piezoelectric effect. Uniform ZnO NRs are coated on PVDF nanofibers, where the nanobrush structure possesses more contact area for organic pollutants. Under both solar and mechanical forces, PZA photocatalyst can rapidly degrade MO in aqueous solution with excellent stability. This proposed PZA nanobrush heterostructure provides a reliable way to enhance the photocatalytic activity, which has infinite potential in the field of environmental protection.

## 2 Experimental section

### 2.1 Synthesis of PZA nanobrush photocatalyst

**Figure 1:**
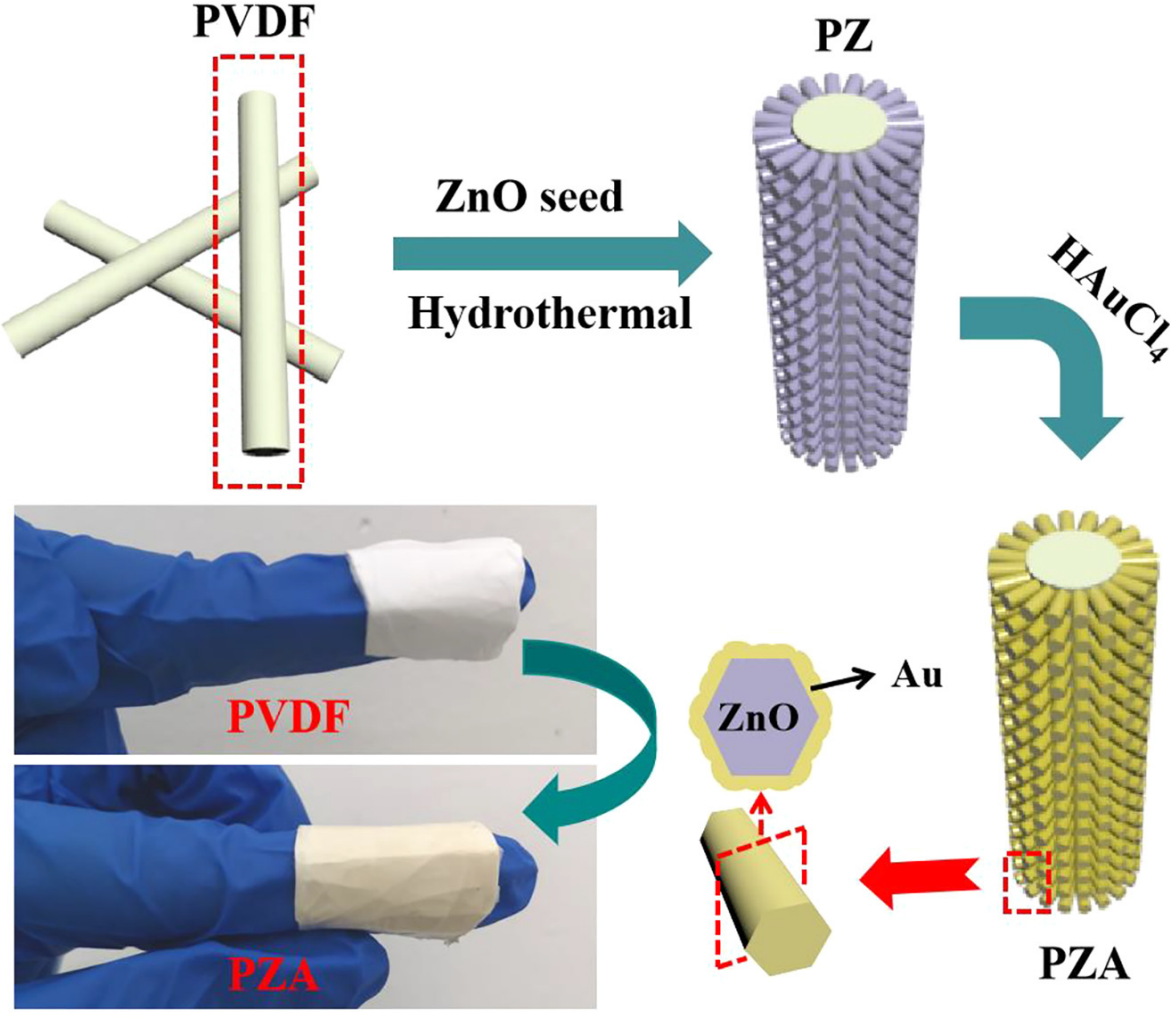
Schematic diagram of the process for the preparation of PZA photocatalyst.

The schematic synthesis process of hybrid PZA nanobrush was shown in Figure 1. PVDF powder (Mw ∼ 275,000, Sigma-Aldrich) was dissolved in mixture of dimethylformamide (DMF) and acetone solution (8:2 v %) at 70 °C to form 15 wt % electrospinning precursor, which was injected into 10 mL syringe with a 22G stainless steel tip needle (connected to the positive electrode) after cooled to room temperature. The substrate of PVDF nanofibers was collected on a nickel (Ni) foil connected to a ground electrode. The distance between needle and collector was 12 cm with applied voltage of 15 kV and the flowrate of 4 mL/h. The electrospinning time was maintained at 20 min.

To grow ZnO NRs around PVDF nanofibers, the ZnO seed layer was firstly deposited on PVDF by the radio frequency magnetron sputtering with 100 W power for 300 s. Then PVDF covered with ZnO seed was immersed into 50 mM hexamethylenetetramine (HMTA) and 50 mM Zn(NO_3_)_2_·6H_2_O solution in Teflon lined stainless steel autoclave at 90 °C for 12 h. Take out the PVDF/ZnO (PZ) nanobrush after cool to room temperature, and wash with ethanol and deionized (DI) water, and then dried at 90 °C in an oven.

Thereafter, the Au was prepared through the catalysis of ZnO NRs under UV irradiation, and the specific reaction process is as follows [21].

$$HAuCl_{4}\to H^{+}+{\left(Au^{3+}Cl_{4}\right)}^{-}$$

$${\left(Au^{3+}Cl_{4}\right)}^{-}\to Cl^{0}+{\left(Au^{2+}Cl_{3}\right)}^{-}$$

$$2{\left(Au^{2+}Cl_{3}\right)}^{-}\to {\left(Au^{3+}Cl_{4}\right)}^{-}+{\left(Au^{+}Cl_{2}\right)}^{-}$$

$${\left(Au^{+}Cl_{2}\right)}^{-}\to Au^{0}+Cl^{0}+Cl^{-}$$

Typically, the PZ nanobrush was immersed into 0.1 mM HAuCl_4_ solution under UV irradiation (365 nm, 300 W) for 10 min to obtain Au on the surface of ZnO NRs. Take it out and wash with ethanol and DI water in turn, and dried at 60 °C in an oven finally forming PZA nanobrush.

### 2.2 Apparatus and characterization

The surface characteristics of prepared photocatalyst were measured by scanning electron microscopy (SEM, ZEISS Sigma500) equipped with an energy dispersive spectrometer (EDS). The crystal phase was recorded by X-ray diffraction (XRD) (SmartLab9), and the elemental compositions were measured by X-ray photoelectron spectroscope (XPS, Thermo Fisher Scientific 250Xi). UV-vis absorbance spectra were carried out in the wavelength range of 200–900 nm at room temperature through an UV-vis-NIR spectrometer. Raman spectra were performed on a Raman spectrometer (Horiba HR Evolution 800) under an excitation laser of 532 nm with power of 0.48 mW. The electrical properties of photocurrent spectra were obtained through a conventional three-electrode system with an electrochemical working station (CHI 760E). Electrochemical impedance spectroscopy (EIS) was carried out in 10 g/L^−1^ NaCl as the electrolyte at open-circuit potentials with a frequency range of 0.1–10^5^ Hz and amplitude of 5 mV. The optical properties of photoluminescence (PL) were measured with a 325 nm He-Cd laser at room temperature (FLS1000).

### 2.3 Piezo-photocatalytic activity measurement

Methyl orange (MO) solution of 20 mg/L was chosen as a pollutant to evaluate the piezo-photocatalytic performance of PZA nanobrush. A simulated solar source was employed via applying a Xe lamp (300 W) equipped without cut-off filter. Before irradiation, the prepared photocatalysts were firstly kept in 50 mL MO solution with concentration of 20 mg/L for 30 min under the dark to reach adsorption–desorption equilibrium. The stir was proceeded in blender with the speed of 800 r/min, and the ultrasound was performed by an ultrasonic machine with power of 200 W. The UV-vis absorption spectra were used for analyzing the degradation efficiency during the catalytic process. The photocatalytic measurements were repeated five times under the same catalytic condition, and extracted the average values to avoid error.

## 3 Results and discussion

**Figure 2:**
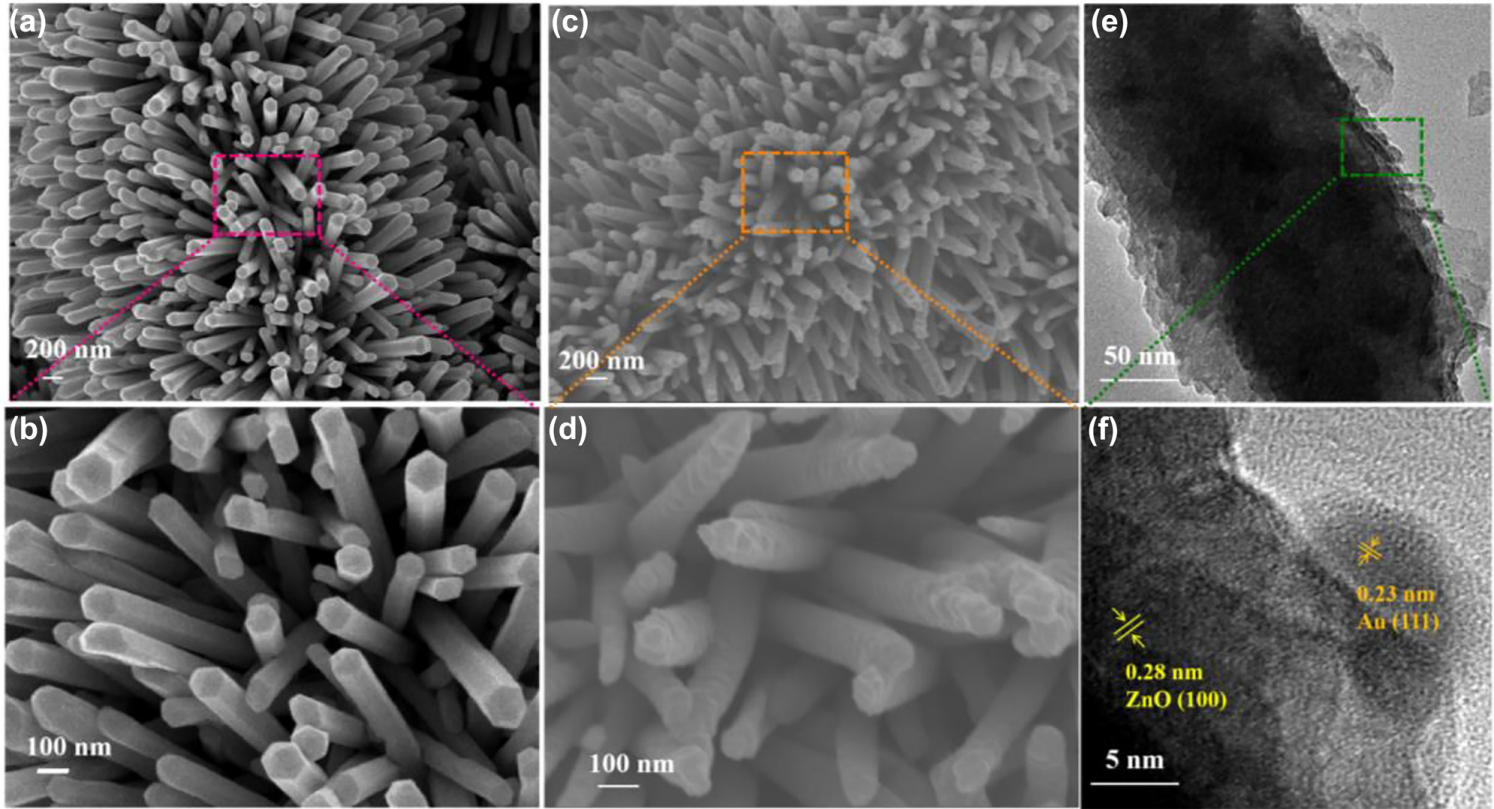
SEM images of (a) PZ nanobrush, (c) PZA nanobrush; (b) and (d) are the enlarged view of PZ nanobrush and PZA nanobrush, respectively. (e) TEM images of the PZA, (f) HRTEM images of the areas indicated in (e).

To reveal the surface morphology of the proposed photocatalysts, the SEM images with different magnifications were characterized shown in Figure 2. The bare PVDF nanofibers presented the hierarchical three-dimensional structure with an uniform diameter of about 2 μm (Figure S1a). As observed in Figure 2(a), the whole surface of PVDF was compactly covered with the ZnO NRs arrays, which were around and uniformly aligned on the nanofiber. From the further amplification in the top view as illustrated in Figure 2(b), the cross-sectional shape of ZnO NRs were hexagonal with an average diameter of 100 nm. After decorated with Au, ZnO NRs were still tightly attached on the surface of PVDF, forming PZA nanobrush structure (Figure 2(c)). It can be obviously observed in Figure 2(d), the dense Au around ZnO NRs possessed rough surface, and the tip becomes sharp due to the chemical reduction. The PZA nanobrush possesses large surface area, which can afford multiple sites for adsorbing surfactant and photocatalytic reaction centers to enhance photocatalytic activity. Figure 2(e) depicted the TEM image of one single ZnO/Au NR, showing the core–shell structure. The whole surface of the ZnO is coated with rough Au, and small AuNPs located at the outer shell. Figure 2(f) identify the high-resolution TEM (HRTEM) images of the ZnO core and Au shells, where the lattice distance of 0.28 nm corresponds to the spacing of (100) plane of ZnO and the interplanar spacing of 0.23 nm corresponds to (111) plane of Au. Furthermore, the EDS elements mapping was measured shown in Figure S1(b), where the Zn, O, Au elements were uniformly distributed along nanofibers indicating that ZnO NRs and Au were successfully synthesized on the PVDF nanofiber.

**Figure 3:**
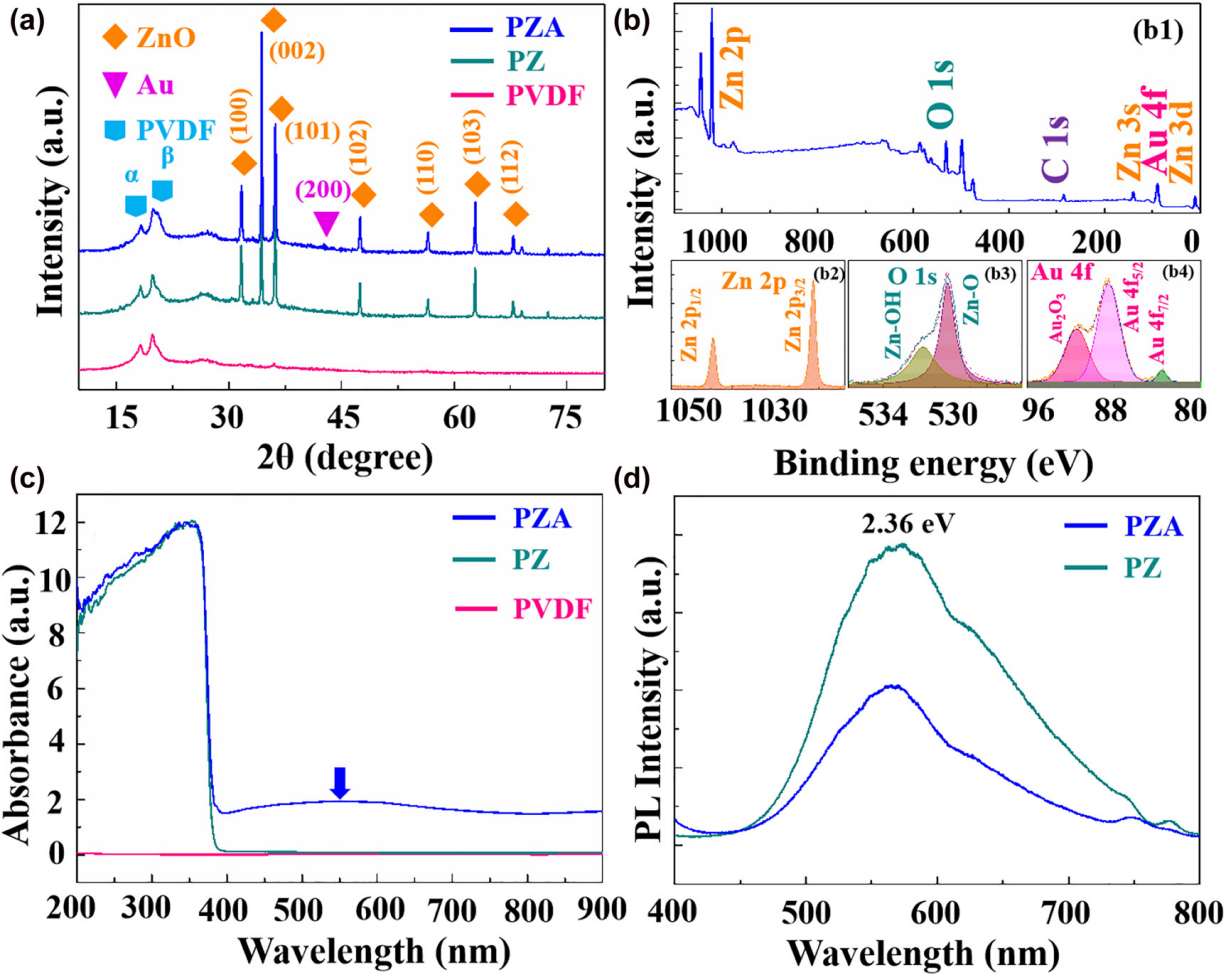
Elemental characterization and optical properties of photocatalysts. (a) XRD patterns of PVDF, PZ and PZA nanostructures; (b) XPS of PZA nanobrush: (b1) the XPS spectra in survey; (b2)–(b4) the high-resolution XPS spectra of Zn 2p, O 1s, Au 4f respectively; (c) UV-vis absorption spectra of PVDF, PZ and PZA nanostructures; (d) PL spectra of PZ and PZA nanobrush.

The crystallographic structures of the prepared catalysts were analyzed by XRD analyses as shown in Figure 3(a), where both the diffraction peaks occurred at 18.3° and 20.0°, respectively, indicating the α phase and β phase of PVDF [22]. The distinct diffraction peaks at 31.6°, 34.4°, 36.2° and 62.8° can be observed, which can be referred to hexagonal ZnO wurtzite structure corresponding to (100), (002), (101) and (103) planes [23], and the weak peak of Au was found at 44.04° of (200) crystal facet duo to a small amount of Au [24]. The XRD analysis revealed that the fabricated ternary PZA catalyst possessed high crystallinity. In addition, the electronic states and chemical compositions of the PZA composites were characterized by XPS spectra. The wide scan spectra of the substrate in Figure 3(b1) clearly manifest that the hybrid nanostructures are involved with Au, Zn, O, and C elements. The high-resolution XPS spectra of Zn 2p, O 1s and Au 4f are shown in Figure 3(b2)–(b4), respectively. The two characteristic peaks at 1021.3 and 1044.3 eV are attributed to Zn 2p_3/2_ and Zn 2p_1/2_, respectively, suggesting the existence of Zn^2+^ (Figure 3(b2)). The peak of O 1s spectra at 530.3 eV is assigned to the lattice oxygen of ZnO crystals [25], and the peak at 531.6 eV corresponding to the oxygen species of hydroxyl groups at the surface of ZnO NRs (Figure 3(b3)) [26]. Moreover, the Au 4f peaks in PZA nanostructures are located at 83.9 and 87.7 eV corresponding to Au 4f_7/2_ and Au 4f_5/2_ of Au, supporting the insertion of crystallized Au [27] and the peak at 91.3 eV arises from the oxidation state of Au^3+^ (Figure 3(b4)). The XPS analysis above further showed that ZnO and Au coexisted in the synthesized photocatalyst.

The UV-vis absorption spectra of PZA, PZ and PVDF nanostructures in range of 200–900 nm are shown in Figure 3(c). The spectrum of bare PVDF has no absorption peak. While after decoration of ZnO NRs, a strong light absorption of PZ presents at the near ultraviolet region owing to wide band gap of ZnO [28]. As expected, PZA heterostructure clearly exhibits a strong absorption in the range of visible light, which confirms that the PZA heterostructure can improve photocatalytic behavior under visible light irradiation. Furthermore, the obvious absorption peak at 550 nm is observed, corresponding to the SPR effect of Au [29]. To elucidate the photogenerated carriers excitation/separation ability, the room-temperature photoluminescence (PL) spectra of the PZ and PZA heterostructures are displayed in Figure 3(d) at the laser wavelength of 325 nm. Obviously, there is a broad emission peak (2.36 eV) of these two substrates in the range of 500–700 nm arising from electron migration from the zinc interstitial/oxygen vacancy to the valence band (VB) [30]. The intensity of PL peak for PZA is significantly lower than that of PZ heterostructures, manifesting a reduced recombination rate of photo-induced electron–hole pairs in the PZA heterostructure, which promotes more significant catalytic efficiency for subsequent MO degradation.

**Figure 4:**
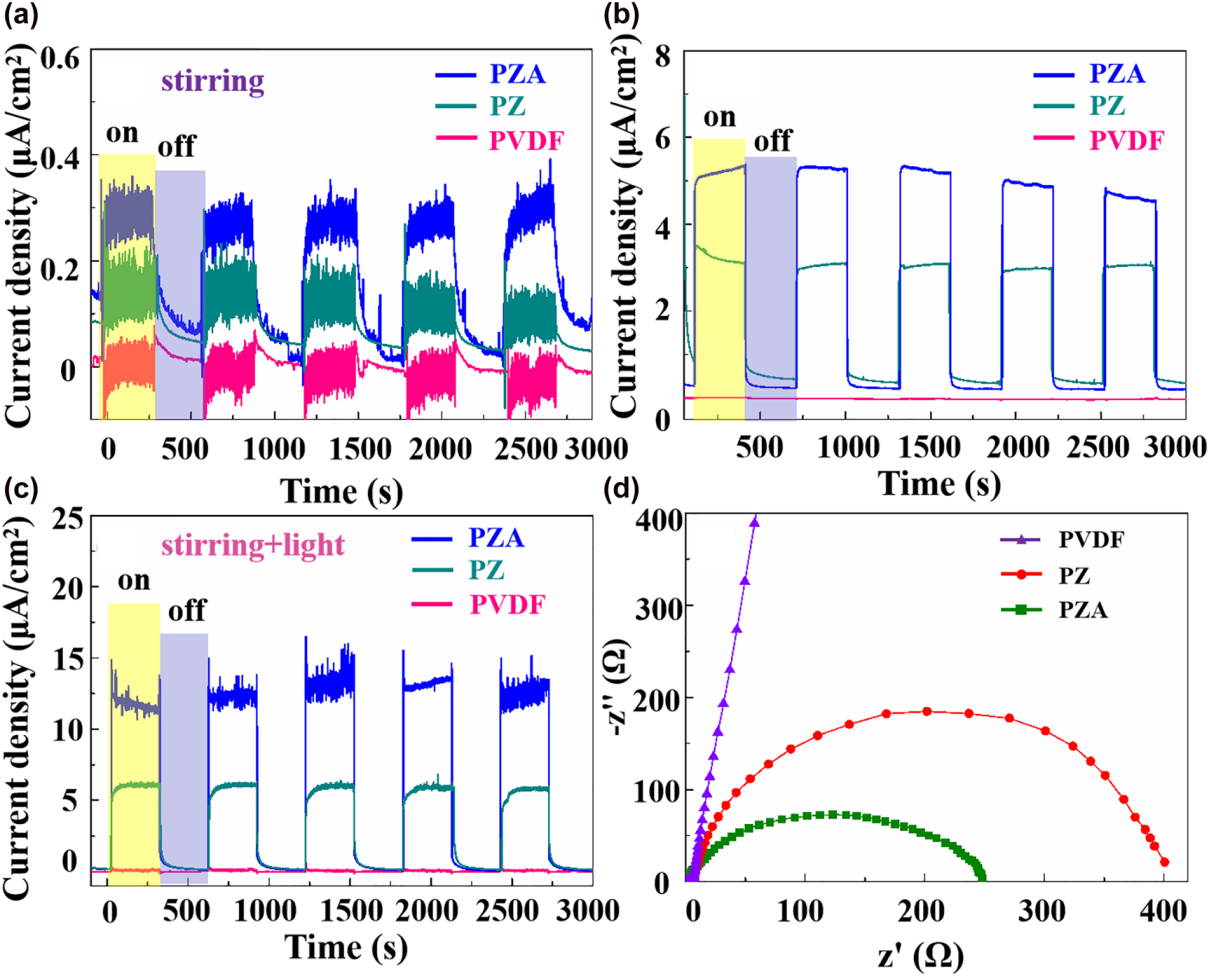
The current signal curves of PZA, PZ and bare PVDF photocatalysts. (a) under stirring, (b) under light, (c) under stirring and light. (d) EIS Nyquist plots of PVDF, PZ and PZA.

Due to the efficiency of electron–hole separation, the photoelectrochemical performances of the photocatalysts can be characterized by the current response, where the higher photocurrent density means the higher carrier density separation efficiency [31]. The prepared photocatalysts on Ni foil served as the working electrode, a Ag/AgCl electrode (KCl saturated) was used as the reference electrode, and a graphite rod was acted as the counter electrode. The current density of PZA, PZ and bare PVDF photocatalysts in the dark under stirring was shown in Figure 4(a) without any bias. When the magnetic stirring was performed at 800 r/min, the current enhanced immediately and stabilized at about 0.3 μA/cm^2^ for PZA photocatalysts, which is higher than that of PZ due to the assistance of Au. With the stirring off, both the currents declined gradually. While for bare PVDF, the signal intensity has slight fluctuations under stirring, lower than that of PZ ascribed to the bi-piezoelectric effect. When a light was applied to photocatalysis, the current density of PZA and PZ increased dramatically and stabilized at about 5 and 3 μA/cm^2^ respectively. Once the light turned off, the signal declined rapidly, and the bare PVDF still has no response to light (Figure 4b). Under synergy of stirring and light, the current density of the three photocatalysts shows the similar trend. Among them, PZA composites present the highest performance at 13 μA/cm^2^, which is much higher than that of PZ at 6 μA/cm^2^. When the magnetic stirring and light were taken away, the current density dropped sharply with time and stabilized close to 0 (Figure 4c). More interesting, the noise of the photocurrent for PZA is larger than that of PZ and PVDF under synergy of stirring and light, proving the PZA is more sensitive to the mechanical vibration, which is beneficial to strong built-in electric field from ZnONRs and PVDF promoting photo-induced charge transport efficiency. For further comparison, a histogram of current density under different conditions was shown in Figure S2, where the synergy of stirring and light exhibits better promoting effect on current response, which can ascribe to the fast separation of photoinduced electrons/holes arising from the piezoelectric field. Therefore, the synergistic effect of bi-piezoelectric field produced by mechanical forces and SPR of Au can effectively enhance the current response of PZA, and further improve photocatalytic efficiency. Moreover, to further analyze carrier separation and transfer efficiency, the electrochemical impedance spectroscopy (EIS) of PVDF, PZ and PZA catalyst was measured shown in Figure 4(d), where the semicircular diameter in the EIS Nyquist diagram represented the charge transfer resistance inversely related to the charge transfer efficiency. It is clear that the semicircle of PZA is much smaller than PZ and PVDF, indicating the smaller charge-transfer resistance and higher interfacial carrier transfer ability for PZA.

**Figure 5:**
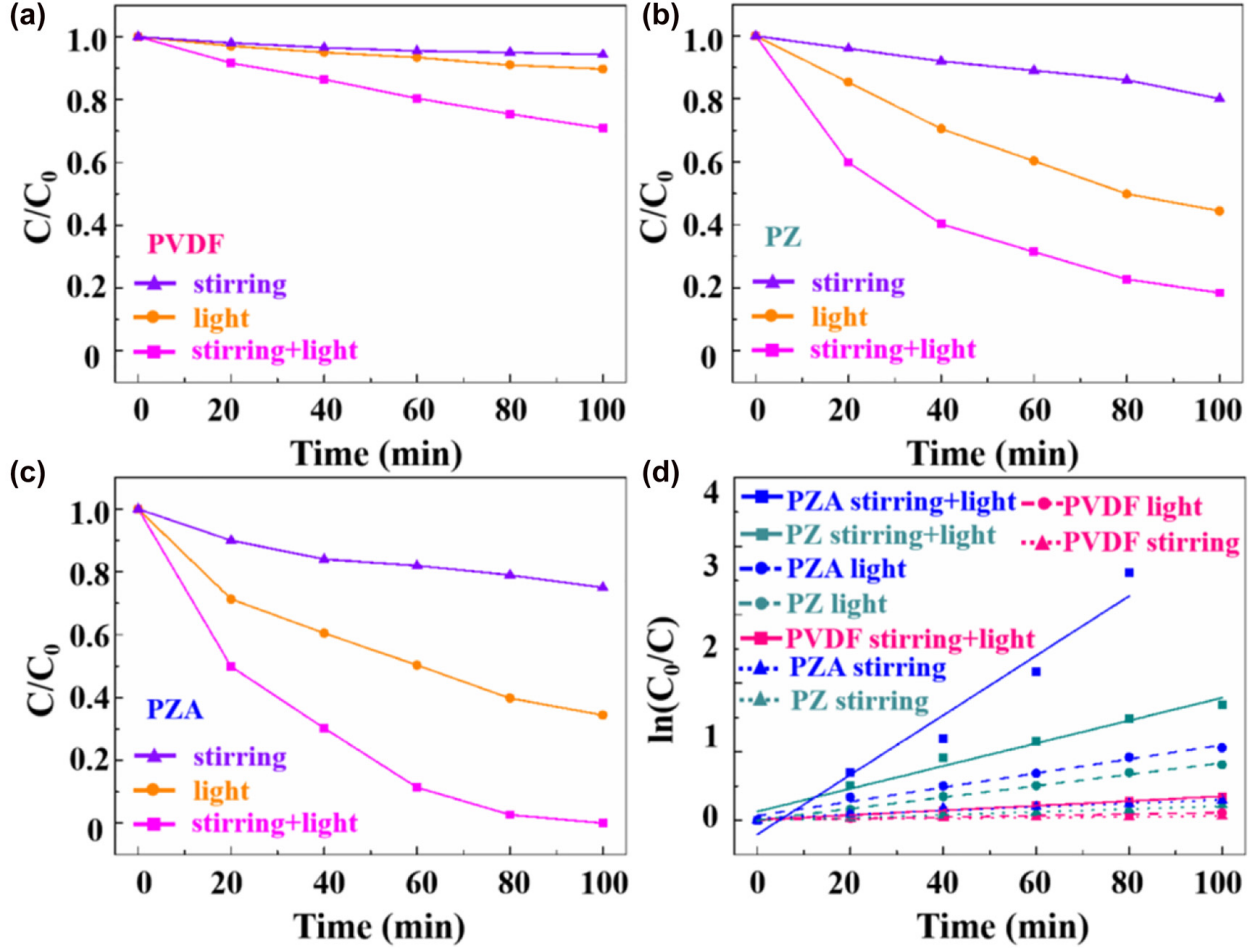
Degradation as a function of irradiation time under different conditions for (a) PVDF, (b) PZ, (c) PZA photocatalysis, (d) catalytic degradation kinetic curves of MO solution with PZA, PZ and PVDF under the different experimental conditions.

To characterize the piezo-photocatalytic activity, the catalytic degradation performance of PVDF, PZ, PZA photocatalysis in MO aqueous solution was evaluated under different conditions illustrated in Figure 5, where *C*/*C*_0_ shows the degradation percentage, *C* represents concentration after degradation and *C*_0_ represents primal concentration. For bare PVDF, the degradation rates of MO are less than 10% under only stirring or light, and still show a negligible activity less than 30% under both stirring and light in 100 min meaning a limited piezo-photocatalytic effect of bare PVDF (Figure 5(a)). The degradation of MO by PZ catalyst under different conditions (stirring alone, light alone, and stirring + light) are performed as displayed in Figure 5(b). The degradation percentage under light is about 50% after 100 min, which is higher than that under stirring of 20%, indicating that mechanical stirring generates slight separation of electrons/holes. The photocatalytic activity under both stirring and light is much higher, and the degradation of MO decreases 80% after 100 min.

To explore the optimized catalytic degradation performance of PZA, the optimization experiments at reaction time with HAuCl_4_ of 5, 10 and 15 min for PZA-5, PZA-10 and PZA-15 were carried out. The experiment results after 100 min catalytic degradation are shown in Figure S3, where the degradation rate of PZA-10 is visibly higher than that of PZA-5 and PZA-15 whatever under stirring, light or synergy of stirring and light. Based on the test, the optimal catalytic degradation performance of PZA is obtained at a reaction time of 10 min (just to simplify, the following PZA is on behalf of PZA-10), which is maintained to further research throughout the following experiments. For a clearer contrast, the detailed degradation process of PZA is presented in Figure 5c, in which the degradation of MO under stirring is still limited, while the concentration decreases to 34% under light representing that the photogenerated electrons/holes play an vital role in degrading MO. Noticeably, the piezo-photocatalytic efficiency of PZA is fairly high under both stirring and light, which almost degraded MO completely after 100 min owing to the fast separation and low recombination of photoinduced electron and hole.

To better compare the photocatalytic activity of these catalysts, the pseudo-first-order model was used to investigate the photocatalytic degradation kinetics of 20 mg/L MO under different conditions in Figure 5(d):

(1) $$\ln\left(\frac{C_{0}}{C}\right)=kt$$

where *k* shows the reaction rate constant (min^−1^), and *t* shows the degradation time [32–34]. Obviously, under the same condition, the PZA heterostructure possesses the highest *k* value (slope) compared with PZ and PVDF. Moreover, the *k* value of PZA under both stirring and light is much higher than those of the others, demonstrating that bi-piezoelectric effect of ZnO and PVDF, as well as SPR effect of Au can effectively improve the photocatalytic activity of PZA nanobrush.

**Figure 6:**
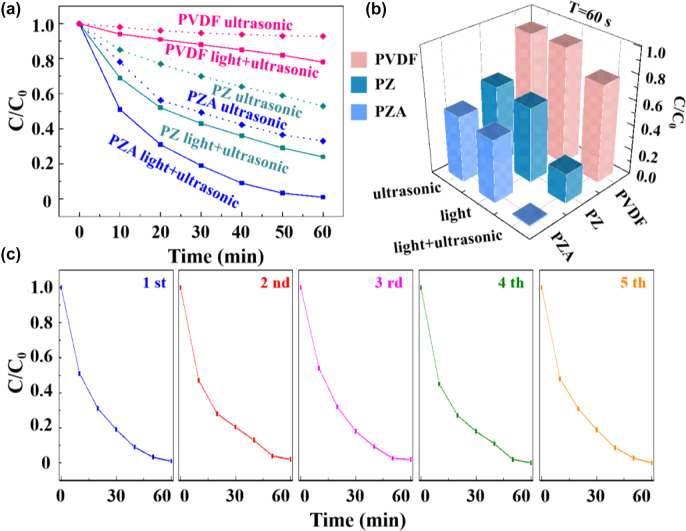
The comparison of degradation efficiency and the stability. (a) Time-dependent degradation efficiency of MO with different catalysts under ultrasonic alone, and both light and ultrasonic; (b) the histogram of degradation in 60 min with the three catalysts under different conditions; (c) the stability of the piezo-photocatalytic activity of PZA nanobrush for degrading MO.

Compared with stirring, ultrasonic can generate stronger built-in electric field with higher power. Figure 6(a) shows the degradation profiles of MO solution by PVDF, PZ and PZA nanostructures under ultrasonic alone and both light and ultrasonic. It can be observed that the degradation of MO almost completes after 60 min for PZA photocatalyst under both light and ultrasonic, which is faster than that under both light and stirring due to the stronger bi-piezoelectric field. To compare directly, Figure 6(b) shows the histogram of degradation after 60 min with PVDF, PZ and PZA nanostructures under different conditions. The degradation rates under only ultrasonic and under only light are equally matched for the same catalyst. While combine ultrasonic and light together, the MO degradation is greatly enhanced, which is ascribed to the bi-piezoelectric effect from the PZ nanobrush and intensive light harvesting profited from Au during the ultrasonic and illumination processes. Furthermore, the PZA nanobrush has the strongest catalytic efficiency, and PZ catalyst shows better catalytic capability than bare PVDF under the same condition ascribed to high electron–hole separation efficiency as well as large specific surface area. The above results represent that the modification of ZnO NRs and Au on PVDF nanofiber can promote the migration of free charges, increase the absorption of light, and ultimately accelerate the rate of catalytic reaction.

The stability without suffering from photocorrosion is a significant parameter for photocatalyst in practical applications. The catalytic degradation performance of PZA nanobrush was evaluated on MO degradation under both light and ultrasonic for five cycles, which were illustrated in Figure 6(c), where the photocatalytic performance of the ternary PZA nanobrush composite is almost unchanged due to the coverage of the Au shells around the surface of ZnO NRs, giving rise to more effective protection of ZnO from oxidation in solution [15, 35].

**Figure 7:**
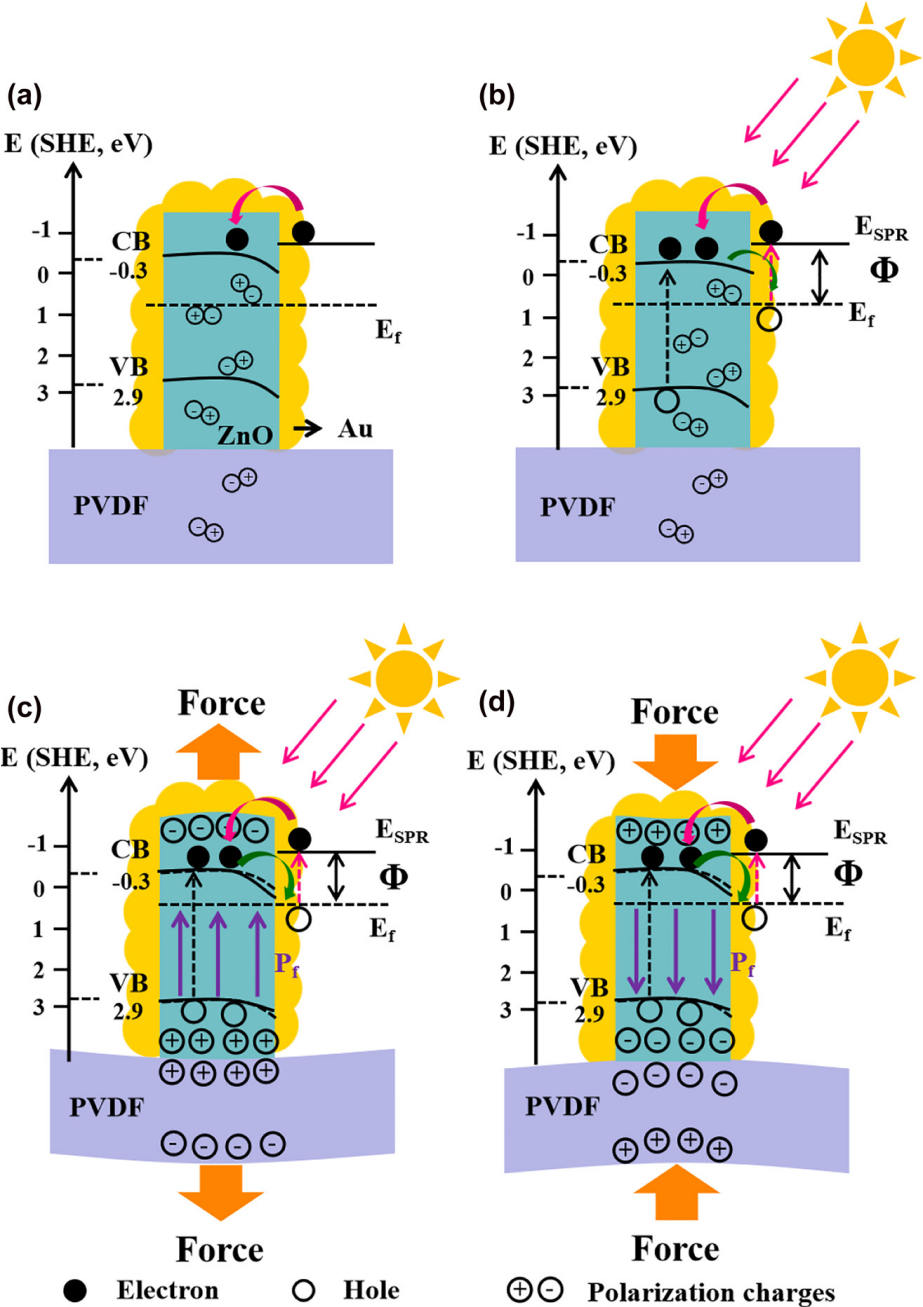
Schematic illustration of photocatalysis mechanism. (a) Without light and ultrasonic, (b) under light alone, (c) under both solar and releasing force, (d) under both solar and pressing force.

Beside on the above results and discussion, the operational mechanism for the piezo-photocatalytic process of PZA nanobrush is shown in Figure 7(a)–(d) through the energy band diagram. The work function of Au is 5.1 eV, which is smaller than ZnO of 5.2 eV. When the Au are coated at the surface of ZnO NRs (Figure 7(a)), the Schottky barrier is formed at their interface, and the electrons transfer from Au to ZnO because electrons shift from the material with lower work function to that with higher work function [36–38]. Under the irradiation of solar light (Figure 7(b)), the electrons in ZnO transfer from VB to conduction band (CB) induced by UV leaving holes with the same numbers at the VB, and the UV-excited electrons would be transferred from CB of ZnO to Au due to Fermi energy level of the heterostructures lower than the energy level of the CB of the ZnO, which can prevent electron–hole recombination. Meanwhile, the photo-induced hot electrons of Au induced by the SPR effect will overcome the Schottky barrier and transfer to the CB of ZnO, leaving the holes to oxidize MO molecule. Besides, the Schottky barrier at the interface of Au and ZnO can further efficiently prevent the injected hot electrons getting back. Under the both solar and mechanical Energy, the built-in bi-piezoelectric field (*P*_f_) is created in ZnO NRs and PVDF nanofibers arising from piezoelectric polarization. At the same time, abundant photo-induced hot electrons are induced by SPR of Au. When the PZA nanobrush is subjected to releasing force (Figure 7(c)), negative charges will produce on the release-bearing surface of ZnO and PVDF, while positive charges will generate on the opposite surface leading to bi-piezoelectric field from ZnO and PVDF. Driven by the built-in bi-piezoelectric field, the photo-induced hot electrons can transfer from Au to CB of ZnO NRs more easily. The transferred electrons reacts with O_2_ to form superoxide radical anion (·O^2−^) and the hole can react with hydroxide ion to generate hydroxyl radicals (·OH), which play a significant role in the degradation of MO molecule [39, 40]. On the contrary, when the PZA nanobrush is occurred by pressing force (Figure 7(d)), the direction of the integrated piezoelectric field will be reversed. Therefore, the combination of bi-piezotronic effect from ZnO and PVDF as well as SPR effect of Au in PZA nanobrush can facilitate the transportation of the charge carriers and suppress the recombination of electron and hole, which will greatly enhance the photocatalytic performance.

## 4 Conclusions

In summary, the ternary PZA nanobrush is successfully synthesized via a facile hydrothermal method and chemical reduction to explore the piezo-photocatalysis activity. It is found that the PZA was demonstrated with much higher piezo-photocatalytic performance for the degradation of MO dye than that of PZ and bare PVDF, which can be ascribed to the bi-piezoelectric effect from the PZ nanobrush and SPR effect profited from Au. Meanwhile, the PZA heterostructures possess the most enhanced catalytic activity under both light and mechanical energy compared with that under light alone and under mechanical energy alone. Furthermore, the PZA nanobrush was found to exhibit faster degradation rate under ultrasonic than that under stirring due to stronger built-in electric field with higher power. These results above represent that the piezo-photocatalysis is an emerging and effective pathway for wastewater treatment, which has great potential in the field of environmental remediation.

## Supplementary Material

Supplementary Material Details
