# Supplementary material for: Plasmonic and bi-piezoelectric enhanced photocatalysis using PVDF/ZnO/Au nanobrush
Source: Nanophotonics. 2022 Jun 10;11(14):3339–49. doi: 10.1515/nanoph-2022-0194 (PMC11501843; doi:10.1515/nanoph-2022-0194)
Supplement: Supplementary file 1 — Supplementary Material Details [file j_nanoph-2022-0194_suppl.doc]

Xiaofei Zhao, Zhen Li, Jing Yu, Chonghui Li, Shicai Xu, Fengrui Li, Chentao Zhang*, Baoyuan Man* and Chao Zhang[[1]](#footnote-2)*

**Plasmonic and** **bi-piezoelectric enhanced** **photocatalysis using PVDF/ZnO/Au nanobrush**


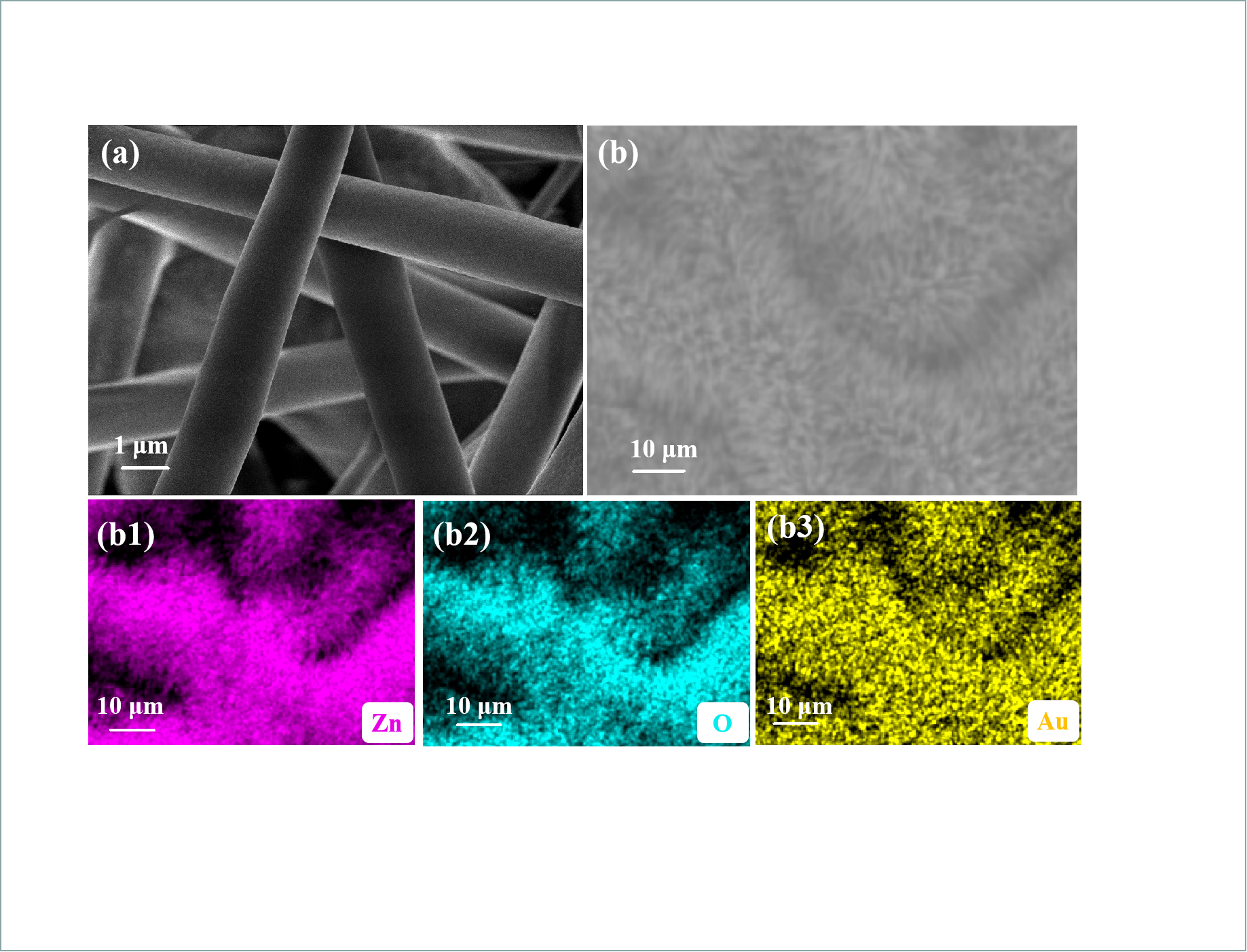


Figure S1. (a) SEM images of PVDF nanofibers, (b) EDS elemental maps from Zn, O, and Au on the PZA nanobrush.


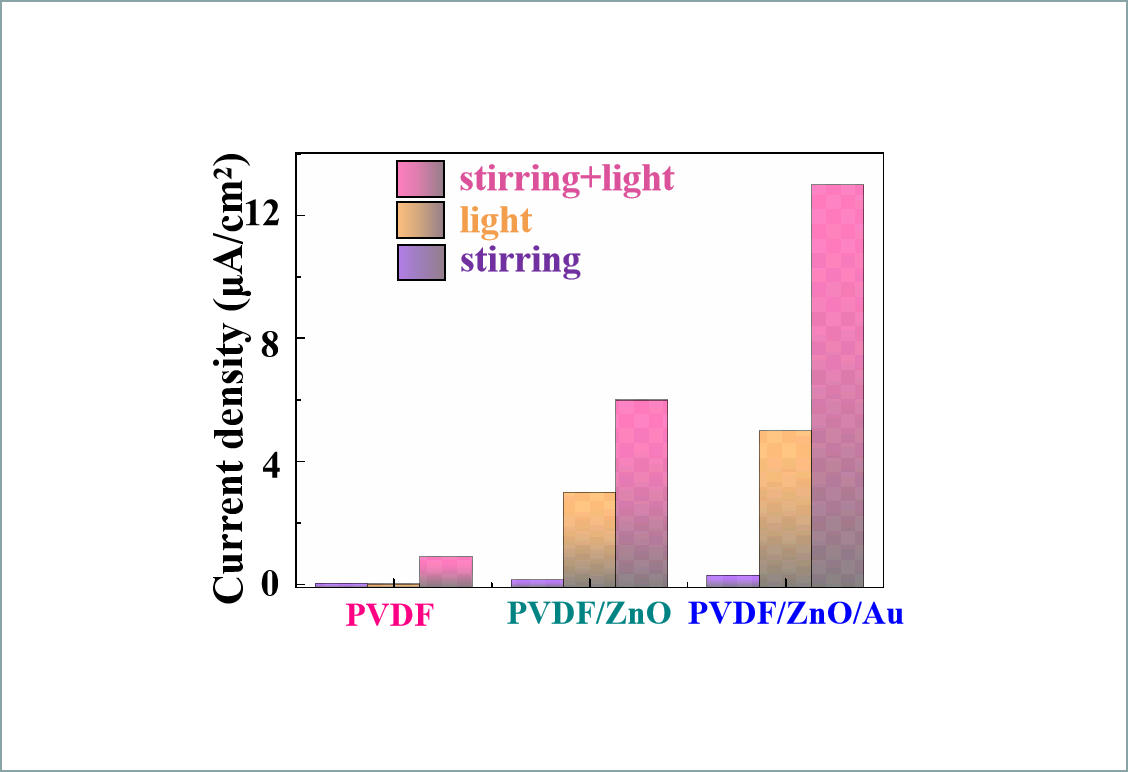


Figure S2. A comparison of current density for PVDF, PZ and PZA under different conditions.


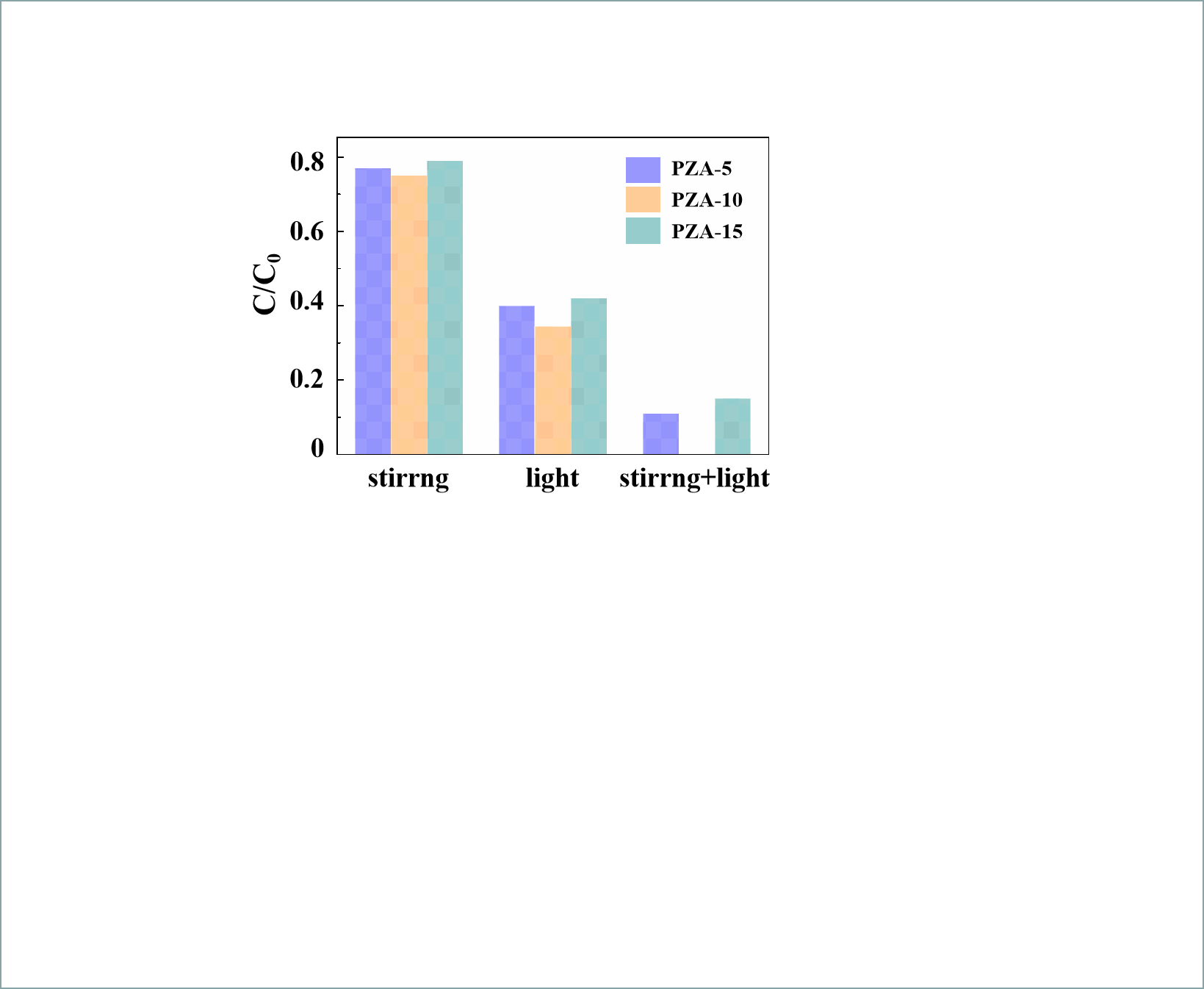


Figure S3. The degradation comparison of PZA-5, PZA-10 and PZA-15 under stirring, light and synergy of stirring and light after 100 min catalytic degradation.

1. ^^*Corresponding author:Chentao Zhang, Baoyuan Man and Chao Zhang,

   mail: [zhangct@xmu.edu.cn,](mailto:zhangct@xmu.edu.cn,) byman@sdnu.edu.cn and [czsdnu@126.com](mailto:czsdnu@126.com)

   Xiaofei Zhao, Zhen Li, Jing Yu, Fengrui Li, Baoyuan Man and Chao Zhang, School of Physics and Electronics, Shandong Normal University, Jinan 250014, China.

   Chonghui Li and Shicai Xu, College of Physics and Electronic Information, Dezhou University, Dezhou 253023, China.

   Chentao Zhang, Department of Instrumental and Electrical Engineering, Xiamen University, Xiamen 361102, China. [↑](#footnote-ref-2)
